# First-trimester proteomic profiling identifies novel predictors of gestational diabetes mellitus

**DOI:** 10.1371/journal.pone.0214457

**Published:** 2019-03-27

**Authors:** Tina Ravnsborg, Sarah Svaneklink, Lise Lotte T. Andersen, Martin R. Larsen, Dorte M. Jensen, Martin Overgaard

**Affiliations:** 1 Department of Clinical Biochemistry and Pharmacology, Odense University Hospital, Odense, Denmark; 2 The Danish Diabetes Academy, Odense University Hospital, Odense, Denmark; 3 Department of Obstetrics and Gynaecology, Odense University Hospital, Odense, Denmark; 4 Department of Biochemistry and Molecular Biology, University of Southern Denmark, Odense, Denmark; 5 Institute of Clinical Research, University of Southern Denmark, Odense, Denmark; 6 Department of Endocrinology, Odense University Hospital, Odense, Denmark; 7 Steno Diabetes Center Odense, Odense University Hospital, Odense, Denmark; Virgen Macarena University Hospital, School of Medicine, University of Seville, SPAIN

## Abstract

**Background:**

Gestational diabetes mellitus (GDM) is a common pregnancy complication associated with adverse outcomes including preeclampsia, caesarean section, macrosomia, neonatal morbidity and future development of type 2 diabetes in both mother and child. Current selective screening strategies rely on clinical risk factors such as age, family history of diabetes, macrosomia or GDM in a previous pregnancy, and they possess a relatively low specificity. Here we hypothesize that novel first trimester protein predictors of GDM can contribute to the current selective screening strategies for early and accurate prediction of GDM, thus allowing for timely interventions.

**Methods:**

A proteomics discovery approach was applied to first trimester sera from obese (BMI ≥27 kg/m^2^) women (*n* = 60) in a nested case-control study design, utilizing tandem mass tag labelling and tandem mass spectrometry. A subset of the identified protein markers was further validated in a second set of serum samples (*n* = 210) and evaluated for their contribution as predictors of GDM in relation to the maternal risk factors, by use of logistic regression and receiver operating characteristic analysis.

**Results:**

Serum proteomic profiling identified 25 proteins with significantly different levels between cases and controls. Three proteins; afamin, serum amyloid P-component and vitronectin could be further confirmed as predictors of GDM in a validation set. Vitronectin was shown to contribute significantly to the predictive power of the maternal risk factors, indicating it as a novel independent predictor of GDM.

**Conclusions:**

Current selective screening strategies can potentially be improved by addition of protein predictors.

## Introduction

Gestational diabetes mellitus (GDM), defined as glucose intolerance diagnosed during pregnancy, is a common complication of pregnancy associated with preeclampsia, caesarean section, macrosomia, neonatal morbidity and future development of type 2 diabetes (T2D) in both mother and child [1–4]. The prevalence of GDM depends on the population and diagnostic criteria used. In Denmark the prevalence is close to 2% by the current diagnostic criteria [5, 6], however, the implementation of the World Health Organzation 2013 (WHO13) guideline is expected to increase the prevalence of GDM in Denmark substantially, posing a major challenge to the healthcare system and economy [7–9].

Obesity is a major risk factor of GDM and obese pregnant women have an up to eight times higher risk of developing GDM compared with normal-weight pregnant women [10]. Thus, the increasing prevalence of overweight and obesity worldwide constitutes a major accelerator of future incidence of GDM. In addition to obesity, a number of well-described risk factors based on maternal characteristics and history are associated with GDM including diabetes in first degree relatives, ethnicity, previous GDM pregnancy and previous macrosomia [11]. Different combinations of the risk factors have been used to develop various prediction models, some of which have been implemented for routine selective GDM screening [12].

The quest for effective screening tools for early and accurate prediction of GDM has intensified within the last decade, as this would create an open window for preventive treatment in terms of lifestyle interventions. To this end, a number of biochemical markers has been investigated for their potential use as biomarkers of GDM, as recently reviewed by Powe [13], however, so far none of them has been proven powerful enough for clinical use in terms of diagnostic sensitivity and specificity. The majority of the studies has centred on individual protein markers of insulin resistance and inflammation such as adiponectin, sex hormone binding globulin (SHBG) and C-reactive protein [14–22], with a few studies also investigating the potential of combining biochemical markers with maternal risk factors [17, 23, 24].

In a recent study, we used targeted mass spectrometry [25] to address the GDM predictive power of a panel of proteins, which have previously been suggested as markers of either GDM or T2D. Here, the multimarker models only marginally improved the performance of adiponectin, indicating a need for identification of new superior markers.

In this present nested case-control study we report the findings of an elaborate proteomics discovery study. A total of 548 proteins were quantitated by shotgun proteomics in first trimester serum samples for identification of potential protein biomarkers, that are completely novel in relation to GDM. A selected number of potential markers was further measured in a second set of samples and evaluated for their individual contributions as predictors of GDM when combined with maternal risk factors. This procedure revealed vitronectin as a novel independent predictor of GDM.

## Methods

### Samples and clinical data

The samples used in this nested case-control study was procured from a biobank of ~20,000 first trimester serum samples collected from the routine screening for Down syndrome at Odense University Hospital (2008–2012) as described in our previous work [25]. All 270 samples included in the current study have been taken between gestational week 8 + 3 days and week 13 + 6 days. Inclusion criteria for patients were; singleton pregnancies, body mass index (BMI) ≥27 kg/m^2^ and HbA_1c_ values of <6.5% (48 mmol/mol) at the time of GDM diagnosis. The BMI was reported at the first ultra sound screening taking place between gestational week 11 + 0 days and week 14 + 0 days and the BMI ≥27 kg/m^2^ cut-off was chosen in accordance with the Danish GDM screening guidelines[11]. The 135 GDM cases were matched to 135 controls based on the year of sampling and BMI. In addition to the already collected clinical data [25], information on the maternal risk factors; previous GDM, family history of diabetes and previous birth of a child with macrosomia, were manually retrieved from patient medical records. All aspects of the study were approved by the local ethics committee (S-20130092) and exemption was given for obtaining written informed consent.

### Discovery proteomics; sample preparation and nanoLC-MS/MS analysis

A proteomics discovery workflow was applied to 60 serum samples (30 GDM cases and 30 controls, selected at random) and a reference pool of first-trimester serum from >10 individuals. Of each sample, 15 μl serum was depleted of the 14 most abundant proteins using an Agilent Human 14 Multiple Affinity Removal Spin Cartridge (Agilent Technologies, Santa Clara, CA, USA) according to the manufactures manual. Of the reference pool 240 μl serum was depleted. The depleted serum samples were switched to 100 mM Triethyl ammonium bicarbonat (TEAB) buffer by spin filtration on Amicon Ultra-4 Centrifugal Filter Device (Merck Millipore Ltd., Cork, Ireland), dried and reconstituted in a fixed volume, 30 μl, 100 mM TEAB prior to denaturation, reduction, alkylation and trypsinization using a protocol modified from Overgaard et al [26], (for detailed description see S1 Supporting Methods). Peptides were purified using Oasis HLB 10 mg cartridges (Waters, Milford, MA, USA), dried and reconstituted in 40 μl 100 mM TEAB. The peptide concentration of each sample was measured on a NanoDrop (Thermo Scientific) and 50 μg was labelled with tandem mass tag (TMT) 10-plex (Thermo Scientific, Waltham, MA, USA) according to the manufactures manual. The amine-reactive TMT 10-plex targets all peptides. It consists of 10 separate isobaric mass tags, which upon fragmentation in MSMS give rise to reporter ions of 10 different masses. Relative quantitation is achieved based on the ion intensity of each of the reporter ions normalized to that of a common reference pool. For each TMT 10-plex experiment 2 replicates of the reference pool, 4 samples from GDM cases and 4 samples from control subjects were labelled. The experiment was repeated 8 times to accommodate all 60 samples. For each TMT 10-plex experiment all samples were pooled in 1:1 ratios and 50 μg was purified on custom made Poros R2/R3 (Thermo Scientific) micro columns, dried, reconstituted and subjected to hydrophilic interaction liquid chromatography (HILIC) fractionation (13–17 fractions) on a TSK Amide-80 3 μm column (Tosoh Bioscience, Stuttgart, Germany) using a Agilent 1200 series high performance liquid chromatography (HPLC) system (Agilent, Santa Clara, CA, US) [27, 28].

The lyophilized fractions were reconstituted in 0.1% Formic acid (FA) and analysed by liquid chromatography tandem mass spectrometry (LC-MS/MS) on an Easy 1000 nano-flow LC/orbitrap Q Exactive HF system (Thermo Scientific) using a custom made 2 column setup (Reprosil-Pur 120 C18-AQ, Dr. Maisch, Ammerbuch-Entringen, Germany), a two hour gradient and a top 20 shot gun proteomics setup. Raw data was exported to proteome discoverer 2.1 (Thermo Scientific) and searched against the Swiss-Prot human database using mascot (1% false discovery rate (FDR) and 5 ppm). For relative quantitation samples were normalized to the average of the 2 replicates of the reference pool included in each TMT 10-plex experiment.

### Targeted proteomics; sample preparation and MRM-MS analysis

For validation by targeted proteomics the remaining 210 samples (105 cases and 105 controls) were subjected to multiple reaction monitoring mass spectrometry (MRM-MS) analysis. Here, only pre-specified peptides were subjected to relative quantitation, by normalization of their MS peak area of selected precursor/product ion pairs to that of the corresponding spiked-in heavy isotope labelled peptides. In short 15 μl of diluted serum, 1:20 in 50 mM ammonium bicarbonate, was denatured, reduced, alkylated and trypsinized essentially as described by Overgaard et al [26] (for detailed description see S1 Supporting Methods). Individually adjusted amounts of 9 heavy isotope labelled standard peptides SpikeTides_L (JPT Peptide technologies, Berlin, Germany), were added to each sample in approximation of a 1:1 ratio to the endogenous light peptides. Peptide purification was done using Oasis HLB 10 mg cartridges (Waters, Milford, MA, USA). Samples were dried, reconstituted in 0.1% FA and run on an Easy-nLC II nano LC system equipped with a 2 column setup (C18, 2 cm, i.d. 100 μm and C18, 10 cm, i.d. 75 μm [Thermo Scientific]). Peptides were eluted with a four-step 60 min gradient of 0.1% FA in acetonitrile at a flow rate of 300 nl/min and analysed on a TSQ Vantage triple quadrupole mass spectrometer, equipped with a Nanospray Flex ion source (Thermo Scientific), in selected reaction monitoring mode. Coefficient of variation (CV) calculations (intra- and inter-assay) were done by including a serum pool in triplicate in the analysis described above. Each triplicate was analysed twice on the nanoLC-MS/MS system. Dilution curves (S1 Fig) for each peptide were made in triplicate by adding different concentrations of the heavy isotope labelled peptide to the same pool of serum which was then processed as described above. The lower limit of quantification for each peptide was derived from the calibration standard curves (S1 Fig and S1 Table). MRM-MS data are represented as the ratio of endogenous light peptide to heavy isotope labelled peptide (S2 Table).

### Data analysis and statistics

Orbitrap Q Exactive HF and TSQ Vantage raw files were processed using proteome discoverer 2.1 (Thermo Scientific) and Pinpoint 1.3 (Thermo Scientific), respectively. Proteomics data was sorted in Excel 2010 and transferred to an SPSS 21.0 (IBM) database, along with the clinical data, for statistical analysis. The statistical tests used were; Mann-Whitney U test, Fisher’s exact test, ROC analysis and binominal logistic regression. A significance level of *p* <0.05 was applied to all statistical tests in this study.

## Results

**Fig 1:**
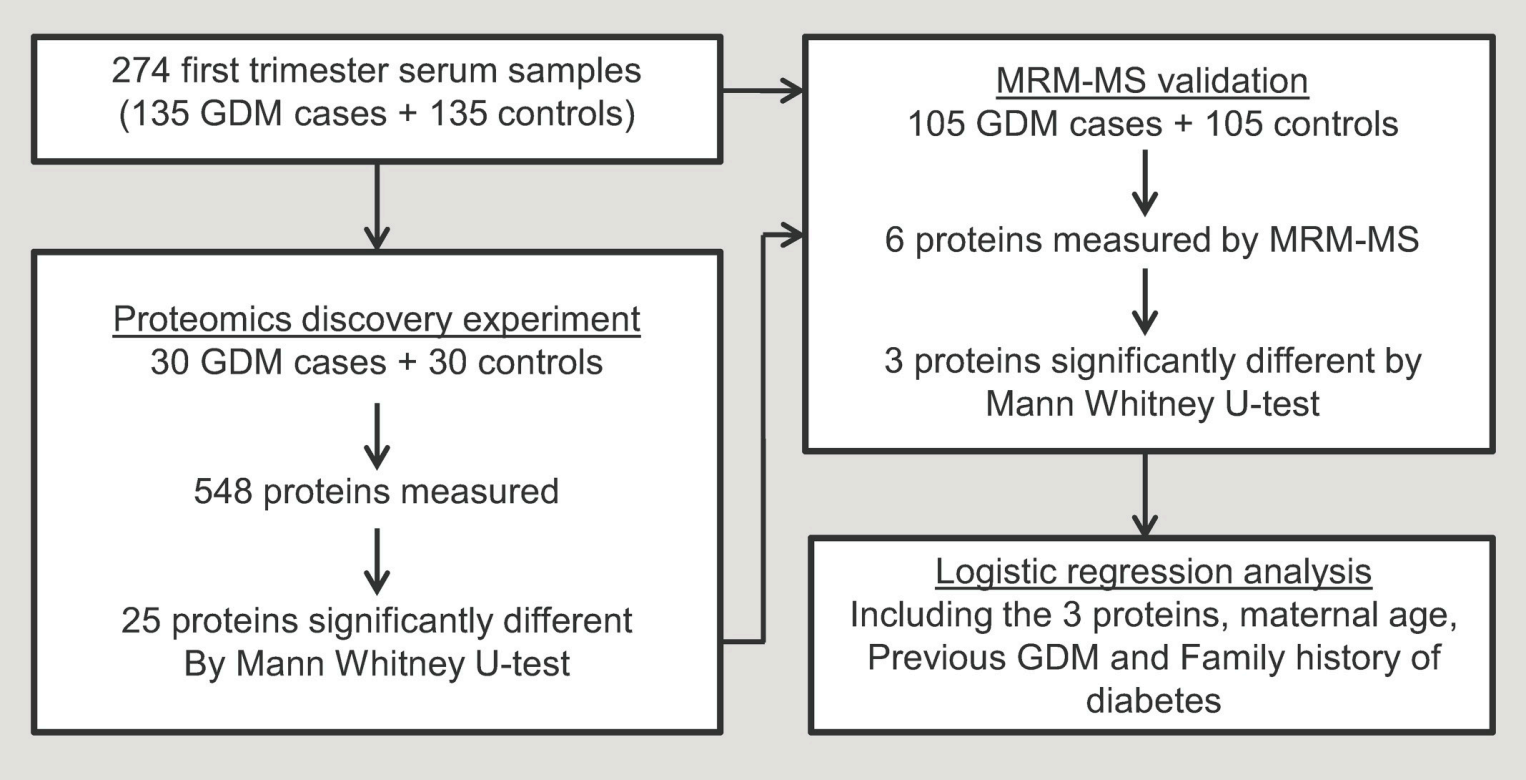
Study workflow. The study comprise two distinct stages; first a proteomics discovery part, for GDM biomarker identification and secondly a MRM-MS validation part.

The experimental setup is illustrated in Fig 1 and consisted of a proteomics discovery part for large scale identification of novel potential protein biomarkers of GDM (*n* = 60) and a targeted proteomics part for validation of selected candidate markers from the discovery part (*n* = 210).

### Clinical data

In addition to the clinical data previously described [25], the maternal risk factors; previous GDM, previous birth of a child with macrosomia and family history of diabetes, were included in this study to assess the individual predictive potential of the protein biomarkers when combined with these predictors (Table 1). In both the discovery and validation set women with GDM gave birth significantly earlier than the control women. This is explained by the routine use of labour induction for women with GDM two weeks before term and translates to the significant lower birth weight and length, and for the discovery set also abdominal circumference. For the validation set, women with GDM were older and had a higher rate of caesarean sections. In both the discovery set and the validation set, there were significantly more women with previous GDM (*p* = 0.025 and *p* = 8.0×10^−6^) and a family history of diabetes (*p* = 0.012 and *p* = 1.3×10^−11^) in the case group as compared to the control group, whereas no such differences were seen for previous macrosomia. As previously reported [25], maternal age was also significantly different between cases and controls for the validation set.

**Table 1:** Clinical data.

	Discovery set			Validation set		
	GDM cases	Controls	*p*	GDM cases	Controls	*p*
*N*	30	30		105	105	
Age at delivery (years)	31.0 (27.0–35.3)	31.0 (29.0–34.0)	NS^a^	32.0 (29.0–36.0)	30.0 (27.0–33.0)	0.001^a^
BMI, week 11–14 (kg/(m^2^))	32.7 (30.9–36.5)	33.0 (31.0–36.0)	NS^a^	32.6 (30.4–36.0)	32.4 (30.2–36.0)	NS^a^
Family history of diabetes	14 (47)	4 (13)^c^	0.012^b^	49 (48)^d^	6 (6)^e^	1.3×10^−11^ ^b^
Previous GDM	6 (20)	0 (0)^c^	0.025^b^	17 (17)^d^	0 (0)^e^	8.0×10^−6^ ^b^
Previous macrosomia	1 (3)	1 (3)^c^	NS^b^	10 (10)^d^	5 (5)^e^	NS^b^
GA at time of sample (days)	74 (68–80)	73 (66–78)	NS^a^	73 (68–79)	72 (69–80)	NS^a^
GA at delivery (days)	268 (263–272)	283 (273–291)	3.0×10^−6^ ^a^	270 (266–278)	281 (273–288)	2.8×10^−11^ ^a^
Induced delivery	8 (27)	13 (43)	NS^b^	62 (60)	55 (52)	NS^b^
Caesarean section	14 (47)	11 (37)	NS^b^	38 (37)	24 (23)	0.035^b^
Preeclampsia	5 (17)	1 (3)	NS^b^	12 (11)	5 (5)	NS^b^
Weight, child (g)	3350 (2773–3680)	3661 (3523–4079)	0.001^a^	3472 (3135–3845)	3590 (3234–3943)	0.031^a^
Length, child (cm)	51(49–52)	53 (51–54)	0.002^a^	52 (50–53)	52 (51–54)	0.026^a^
Abd. Circ. child (cm)	33(31–34)	34 (33–36)	0.033^a^	34 (32–35)	34 (32–35)	NS^a^
LGA	5 (17)	7 (23)	NS^b^	24 (23)	17 (16)	NS^b^

Data are median and interquartile range or n (%), NS: non-significant, GA: gestational age, Abd. Circ.: Abdominal Circumference, LGA: large for gestational age (>90^th^ percentile).

^a^ Mann-Whitney U test

^b^ Fisher’s exact test

Missing data for

^c^ 3 controls

^d^ 2 cases and

^e^ 7 controls

### Identification and validation of novel potential protein biomarkers of GDM

The proteomics discovery approach, utilizing TMT labelling, was applied to individual first trimester serum samples from 30 GDM cases and 30 control patients. Initially 1015 proteins were identified (1% FDR, 5 ppm), amounting to 548 proteins with a mascot score above 22, two or more unique peptides and presence in more than 73% of the samples. The relative serum levels of these 548 proteins were subjected to Mann Whitney U testing and receiver operating characteristic (ROC) analysis, revealing a significant difference between GDM cases and controls for 25 proteins (Table 2). None of the significant markers remained so after FDR correction by the Benjamini-Hochberg method for 548 variables. The best performing single protein was secreted phosphoprotein 24 with an uncorrected *p*-value of 0.0003 and an area under the curve (AUC) of 0.770.

**Table 2:** Protein serum levels of significant difference from proteomics discovery experiment.

Protein name	GDM cases (*n* = 30)	Controls (*n* = 30)	*p*^a^	AUC^b^ (95% CI)
Secreted phosphoprotein 24	0.83±0.11	1.00±0.20	0.0003	0.770 (0.649, 0.891)
Antithrombin-III	0.90±0.07	0.96±0.07	0.0011	0.744 (0.618, 0.871)
Desmoglein-2	0.93±0.09	1.00±0.11	0.0065	0.704 (0.572, 0.836)
78 kDa glucose-regulated protein	0.95±0.09	1.00±0.08	0.0097	0.694 (0.559, 0.829)
Carboxypeptidase N catalytic chain	0.94±0.12	1.03±0.13	0.0097	0.694 (0.561, 0.828)
Adiponectin	0.80±0.18	0.88±0.14	0.0097	0.694 (0.558, 0.831)
Cystatin-M	1.02±0.18	0.92±0.18	0.0153	0.682 (0.544, 0.820)
Suprabasin	1.01±0.18	0.92±0.20	0.0160	0.681 (0.542, 0.820)
Afamin	1.06±0.13	0.99±0.15	0.0187	0.677 (0.538, 0.815)
Adhesion G-protein coupled receptor G6	0.91±0.15	0.99±0.18	0.0224	0.672 (0.531, 0.812)
Ferritin light chain^c^	1.21±0.27	1.05±0.28	0.0232	0.683 (0.537, 0.830)
Platelet glycoprotein V	1.00±0.11	1.07±0.11	0.0256	0.668 (0.531, 0.804)
IgGFc-binding protein	1.15±0.30	1.02±0.17	0.0266	0.667 (0.527, 0.806)
Vitronectin	1.03±0.07	1.00±0.07	0.0266	0.667 (0.526, 0.807)
Serum amyloid P-component	1.17±0.18	1.06±0.17	0.0266	0.667 (0.528, 0.806)
Receptor-type tyrosine-protein phosphatase S	0.97±0.07	1.02±0.08	0.0266	0.667 (0.529, 0.804)
Connective tissue growth factor	0.91±0.10	1.00±0.17	0.0266	0.667 (0.530, 0.804)
Sex hormone-binding globulin	0.71±0.15	0.82±0.20	0.0298	0.663 (0.526, 0.801)
ADAMTS-like protein 2	1.04±0.18	0.95±0.10	0.0371	0.657 (0.516, 0.797)
Serine protease inhibitor Kazal-type 5	1.04±0.15	0.96±0.13	0.0371	0.657 (0.518, 0.796)
Phospholipid transfer protein	0.98±0.18	1.04±0.14	0.0413	0.653 (0.512, 0.795)
Putative pregnancy-specific beta-1-glycoprotein 7^c^	2.78±2.75	1.23±1.19	0.0422	0.664 (0.515, 0.813)
Platelet glycoprotein Ib alpha chain	0.92±0.16	1.00±0.14	0.0444	0.651 (0.512, 0.790)
Cathepsin Z	1.06±0.14	0.99±0.16	0.0444	0.651 (0.509, 0.793)
Carboxypeptidase N subunit 2	0.95±0.12	1.01±0.12	0.0493	0.648 (0.506, 0.789)

Proteomics data is presented as the ratio between individual samples and the common reference pool. Data are mean ± SD.

^a^Mann Whitney U test

^b^AUC of ROC analysis

^c^Data from 26 GDM cases and 26 controls

In order to explore the putative predictive potential of the protein markers identified by the proteomics discovery approach, an MRM-MS assay was developed, ultimately capable of measuring the relative serum levels of 6 of the original 25 proteins (see S1 Supporting Methods, S1 and S2 Tables, S1 and S2 Figs). The 6-plex assay was applied to the 210 remaining validation serum samples and here the levels of the three proteins; afamin, serum amyloid P-component (SAMP) and vitronectin could be confirmed as significantly different between GDM cases and controls (Table 3).

**Table 3:** Protein serum levels measured by MRM-MS in the validation set.

Protein name	GDM cases (*n* = 105)	Controls (*n* = 105)	*p*^a^	Model 1 *p*^*b*^	Model 2 *p*^*c*^	Model 3 *p*^*d*^
Afamin	2.20±0.37	2.07±0.32	0.005	0.007	NS	NS
Antithrombin-III	0.64±0.10	0.64±0.10	NS	NS	NS	NS
Carboxypeptidase N subunit 2	1.46±0.41	1.50±0.49	NS	NS	NS	NS
Phospholipid transfer protein	1.17±0.17	1.16±0.18	NS	NS	NS	NS
Serum amyloid P-component	2.85±0.58	2.67±0.52	0.042	0.017	NS	NS
Vitronectin	3.03±0.45	2.83±0.48	0.002	0.002	0.046	0.046

MRM-MS data is presented as the ratio of endogenous light peptide to heavy isotope spiked-in peptide

Data are mean ± SD. Significance of contribution to model when combined with ^b^, ^c^ or ^d^ by logistic regression. Models including FamDiab comprise *n* = 201 due to missing data.

^a^Mann Whitney U test

^b^Maternal age (Model 1)

^c^Family history of diabetes (Model 2)

^d^Combined (Model 3)

### Logistic regression of protein biomarkers and clinical risk factors

To further evaluate the biomarker potential of afamin, SAMP and vitronectin as compared to maternal age and the maternal risk factors currently used in routine GDM screening, a number of binominal logistic regression analyses were performed using the data from the validation set (*n* = 210). Previous macrosomia was excluded from this analysis as it showed no significant difference in the univariate analysis, BMI was also excluded as cases and controls were matched on this parameter. The logistic regression analyses were evaluated by ROC analysis and are listed in Table 4 and S3 Table (extended).

**Table 4:** ROC analysis of multivariate models.

Model	Variables included in model	Validation set (*n* = 210)	
		AUC	*p* of AUC
1	Mage	0.638 (0.563, 0.714)	0.001
2	FamDiab	0.707 (0.635, 0.780)	3.9×10^−7^
3	Mage + FamDiab	0.798 (0.736, 0.861)	2.7×10^−13^
A	SAMP	0.581 (0.504, 0.658)	0.042
B	Afamin	0.612 (0.536, 0.689)	0.005
C	Vitronectin	0.625 (0.550, 0.701)	0.002
1.A	Mage + SAMP	0.659 (0.585, 0.733)	6.8×10^−5^
1.B	Mage + afamin	0.676 (0.603, 0.748)	1.1×10^−5^
1.C	Mage + vitronectin	0.681 (0.609, 0.753)	6.0×10^−6^
2.C	FamDiab + vitronectin	0.760 (0.695, 0.826)	1.8×10^−10^
3.C^a^	Mage + FamDiab + vitronectin	0.806 (0.746, 0.867)	6.3×10^−14^

All variables contributes significantly to the model (*p* <0.05). Models including FamDiab comprise *n* = 201 due to missing data.

^a^Model also achieved by logistic regression of the 6 variables; Previous GDM, Family history of diabetes (FamDiab), Maternal age (Mage), afamin, vitronectin and SAMP, removing the variable with least contribution to the model until all remaining variables contributed significantly

When combining family history of diabetes, previous GDM and maternal age in multivariate models, the predictor previous GDM no longer contributed significantly and therefore it was omitted from the remaining analyses (S3 Table). Consequently, each of the three proteins were assessed for their individual contribution as predictors of GDM by combining them with maternal age (model 1), family history of diabetes (model 2) or both (model 3), Tables 3 and 4. When combined with maternal age alone all three proteins contributed significantly to the model. In addition, vitronectin remained a significant contributor when combined with family history of diabetes alone or together with maternal age. Taken together this indicates that vitronectin is an independent predictor of GDM despite the very modest increment in model AUC (0.806 versus 0.798). Finally, we performed a logistic regression analysis, initially comprising all 6 variables; previous GDM, maternal age, family history of diabetes, afamin, SAMP and vitronectin, with stepwise removal of the variable of least significant contribution until all remaining variables contributed significantly. This resulted in the same model comprising only maternal age, family history of diabetes and vitronectin (AUC = 0.806).

## Discussion

Here we have used proteomic profiling of first-trimester sera from obese women with or without GDM to obtain a catalogue of novel biomarker candidates. Of the 548 proteins originally quantified in the discovery study, 25 were identified as significantly different between cases and controls. Six of these were further measured in the validation set where afamin, SAMP and vitronectin could be confirmed as potential predictors of GDM, with only vitronectin adding significantly to the maternal risk factors already used for routine screening. Afamin has recently been associated with GDM by Tramontana et al [29, 30], whereas vitronectin, to our knowledge, is new in this aspect. Higher blood levels of vitronectin have been associated with the risk of metabolic syndrome and T2D [31], making it a plausible candidate biomarker of GDM. Increased levels of vitronectin have previously been identified as a risk factor of preeclampsia [31, 32] and preeclampsia could thus be considered as a potential confounder in our study. However the number of patients with preeclampsia was not significantly different between our case and control group, also; when removing the individuals with preeclampsia from the data set (*n* = 12 + *n* = 5), vitronectin remained a significant contributor in all of the models 1, 2 and 3 with *p* = 0.002, *p* = 0.040 and *p* = 0.046 respectively.

As for the credibility of the discovery study, it is noteworthy that adiponectin and SHBG were among the 25 proteins showing initially significantly different levels by the Mann Whitney U test (Table 2). Both proteins have been intensively investigated as biomarkers of GDM [15, 20, 21, 33] and in our previous study, we found adiponectin and SHBG to be significantly different between GDM cases and controls in the same cohort using different assay methods [25]. Furthermore, the confirmation of afamin, SAMP and vitronectin as novel predictors of GDM, by validation with a different method in a second set of samples, also support the validity of the discovery data as a source of potential GDM markers, despite the lack of significant FDR corrected *p*-values. By contrast, the fact that three proteins could not be confirmed as markers of GDM in our validation set, further emphasize the requirement for subsequent verification in omics-type studies. Due to the sensitivity limit of targeted MS, we were unable to verify the top candidate secreted phosphoprotein 24 (SPP24) using the multiplex validation assay. Further studies are underway to address the potential of this candidate in GDM prediction.

To our knowledge the work presented in this study is the most comprehensive proteomics-based study in GDM so far. In our biomarker workflow we have performed in depth serum proteomic profiling by depletion of the 14 most abundant serum proteins, TMT-labelling, HILIC fractionation and orbitrap-based LC-MS/MS. By using a fairly large number of individual samples, as opposed to pooled samples [34] or very small (n<10) sample size [35], the discovery study allowed for the display of the inter-individual proteome differences and thereby enabled the identification of protein biomarkers associated with small but consistent significant differences between cases and controls. Likewise, the inclusion of maternal risk factors allowed us to better evaluate the actual contribution of the identified protein predictors in comparison to the GDM screening strategy already clinically employed. In our previous study [25] we have already shown that maternal age could aid in discriminating GDM cases from controls and suggested that it should be included in the Danish screening strategy. This suggestion is made further relevant by the present study where the maternal risk factors have been included and maternal age has been shown to be an independent discriminator, adding to the predictive power of the maternal risk factors separately or combined.

As for the limitations of our study; a well-known weakness of the nested case-control study design, is the potential inflation of biomarker performance, as compared to population-based studies. This however, is a compromise that must be made to better accommodate the rather laborious analytical protocols. In our proteomics workflow, the use of immuno-affinity depletion of the most abundant serum proteins is a known source of experimental bias. While the method allows for a more in depth proteomic profile (more proteins identified), it will also inadvertently remove some untargeted low abundant proteins due to unspecific co-depletion.

While evidence based strategies of GDM screening, diagnosis and treatment continue to be highly debated [36, 37] a number of clinical applications for early pregnancy blood-based biomarkers are now emerging. Firstly, universal screening based on a fasting or non-fasting oral glucose challenge test is cumbersome for both patients and healthcare providers and it is performed late in pregnancy. Thus, the benefits of early blood-based screening in combination with risk factors could pave the way for a much better alternative; not only for GDM risk stratification, but also for stratification of GDM-related outcomes such as macrosomia (large for gestational age), preterm birth, caesarean section, hypertensive disorders and shoulder dystocia. Secondly, in Denmark, where a selective screening model based on maternal characteristics and history are recommended, as much as 40% of the pregnant women are referred to oral glucose tolerance test (OGTT) testing, implying the need for a more specific screening model. To this end, addition of one or more biomarkers seems attractive and adaptable to a clinical setting.

In conclusion, this study has provided a comprehensive overview of potential serum protein biomarkers for early GDM prediction and identified vitronectin as a novel independent predictor. Evidently, multivariate models using maternal risk factors in combination with biochemical predictors may improve the discriminative power compared to risk factors alone. However, the GDM field still awaits the magic bullets to appear from biomarker research; a quest that is long but exiting and driven by novel technological developments.

## Supporting information

S1 Supporting Methods (PDF)

S1 Fig Linearity of dilution of stable isotope labelled standard peptides. (PDF)

S2 Fig Linear correlation of tryptic peptides for quantitation of afamin, vitronectin and CPN2. (PDF)

S1 Table MRM 6-plex assay. (PDF)

S2 Table MRM 6-plex assay ratio data. (XLSX)

S3 Table Binominal logistic regression analysis. (PDF)
