# Supplementary material for: First-trimester proteomic profiling identifies novel predictors of gestational diabetes mellitus
Source: PLoS One. 2019 Mar 27;14(3):e0214457. doi: 10.1371/journal.pone.0214457 (PMC6436752; doi:10.1371/journal.pone.0214457)
Supplement: S1 Supporting Methods — (PDF) [file pone.0214457.s001.pdf]

S1 Supporting methods - Ravnsborg et al.

Discovery proteomics; Digestion and TMT labelling of depleted serum samples

Depleted serum samples (originally 15 µl undepleted serum) was reconstituted in 30 µl 100 mM Triethyl ammonium bicarbonat (TEAB) and denatured by addition of an equal amount of 100% trifluoroethanol (TFE) for 1 h at 65°C (protocol adjusted from [1]). Samples were reduced at a final concentration of 7 mM dithiothreitol (DTT) for 30 minutes at 65°C and alkylated by 20 mM iodoacetamide (IAA) for 30 minutes at room temperature. Following a 1:5 dilution in 100 mM TEAB the samples were digested by addition of 3.5 µg trypsin (Sigma, St. Louis, MO, USA) and incubation at 37°C. The process was stopped after 20-21 hours by addition of formic acid (FA) to a final concentration of 1%. The samples were then purified on Oasis HLB 10 mg cartridges (Waters), dried and reconstituted in 40 µl 100 mM TEAB. The peptide concentration of each sample was measured on a NanoDrop (Thermo Scientific) and 50 µg was labelled with 0.4 mg Tandem mass tag (TMT) 10-plex label (Thermo Scientific, Waltham, MA, USA) according to the manufactures manual. For each TMT 10-plex experiment 2 replicates of the reference pool was labelled with the 126 and 131 tag, 4 samples from GDM cases were labelled with the 127N, 127C, 128N and 128C tag and 4 samples from control subjects were labelled with the 129N, 129C, 130N and 130C tag. The experiment was repeated 8 times to accommodate all 60 samples and for each TMT 10-plex experiment all 8 samples and 2 reference pool replicates were combined in 1:1 ratios.

Discovery proteomics: HILIC fractionation and nano LC-MS/MS analysis

For each combined TMT 10-plex experiment 50 µg was purified on custom made Poros R2/R3 (Thermo scientific) micro columns, dried and reconstituted in 45 µl 90% acetonitrile, 0.1% trifluoroacetic acid (TFA). The samples were subjected to hydrophilic interaction liquid chromatography (HILIC) fractionation on a TSK Amide-80 3 µm column, 450 µm OD x 320 µm ID x 17 cm, (Tosoh Bioscience, Stuttgart, Germany) using a Agilent 1200 series HPLC system (Agilent, Santa Clara, CA, US), buffer A being 0.1% TFA and buffer B being 90% acetronitril (ACN), 0.1% TFA. The gradient was set to 100% buffer B to 60% buffer B in 30 minutes at a flowrate of 6 µl/min, resulting in 13-17 fractions after pooling of fractions with a low absorbance [2,3]. The dried down fractions were reconstituted in 0.1% FA and analysed on an Easy 1000 nano-flow LC/orbitrap Q Exactive HF system (Thermo Scientific) using a custom made 2 column setup (Reprosil-Pur 120 C18-AQ, Dr. Maisch, Ammerbuch-Entringen, Germany), a 134 minute gradient and a top 20 shot gun proteomics setup. The gradient of buffer B (100% ACN, 0.1% FA) consisted of five steps: 1→3% B in 3 minutes, 3→25% B in 110 minutes, 25→45% B in 10 minutes, 45→100% B in 3 minutes and 100% B for 8 minutes, the flowrate was set to 250 nl/min.

Targeted proteomics; sample preparation and MRM-MS analysis

The 210 serum samples (105 cases and 105 controls) to be used for validation by MRM-MS analysis were randomized into batches of 20 samples (10 cases and 10 controls) for reasons of practicality. Each batch also included three replicates of a reference serum pool (>10 individuals) used for intra and inter CV calculations.

Sample preparation for MRM-MS analysis was done essentially as described by Overgaard et al [1]. 15 µl of diluted serum, 1:20 in 50 mM ammonium bicarbonate (ABC), was denatured with an equal

volume of 100% TFE, reduced by a final concentration of 7 mM DTT for 30 minutes at 65°C and alkylated by 20 mM IAA for 30 minutes at room temperature. Following a 1:5 dilution in 25 mM ABC the samples were digested by addition of 2.5 µg trypsin (Sigma) and incubation at 37°C. The process was stopped after 20-21 hours by addition of 10% FA to a final concentration of 1%. Individually adjusted amounts of 9 heavy isotope labelled standard peptides (+8 Da or +10 Da), SpikeTides^{TM}_L (JPT Peptide technologies, Berlin, Germany), were added to each sample in approximation of a 1:1 ratio to the endogenous light peptides. Peptide purification was done using Oasis HLB 10 mg cartridges (Waters, Milford, MA, USA), applying 50% ACN for elution of peptides. Samples were dried, reconstituted in 0.1% FA and run on an Easy-nLC II nano liquid chromatography (LC) system equipped with a 2 column setup (C18, 2 cm, i.d. 100 µm and C18, 10 cm, i.d. 75 µm (Thermo Scientific)). Peptides were eluted with a four-step 60 min gradient of buffer B;100% ACN, 0.1% FA (5→10% B in 5 min, 10→33% B in 43 min, 33→100% B in 4 min and 100% B for 8 min), at a flow rate of 300 nl/min and analysed on a TSQ Vantage triple quadrupole mass spectrometer, equipped with a Nanospray Flex ion source (Thermo Scientific), in selected reaction monitoring mode.

MRM-MS assay development

For each of the 25 protein found to be significantly different between GDM cases and controls, prior to false discovery rate correction, a maximum of 10 peptides were selected for MRM-MS assay development. Peptides were selected according to the following criteria; tryptic cleavage, length between 7 and 25 amino acids and calculated hydrophobicity between 12 and 45. Peptides were excluded based on; missed cleavages, NXS/T motives (N-linked glycosylation site), methionines (oxidation), RP or KP motif (no cleavage), serine and threonine rich areas (potential O-linked glycosylation sites) and any post translational modifications sites listed in the UniProtKB datasheet of

the protein. All peptides were ascertained to be unique by BLASTP analysis against the humane proteome. Transitions were set to precursor ions of charge state +2 and product ions of charge state +1, with a mass to charge ratio <1500. Only y-ions >y3 was included, with b-ions only being tested in case of y-ions not giving any results. The MRM-MS assay was tested on a pool of first trimester serum and correct peptide identities were confirmed by the coelution and identical transition patterns of spiked-in isotopically heavy labelled standard peptides. The performance and reproducibility of each transition was manually assessed followed by collision energy optimization. The final assay comprised 9 (18) peptides representing 6 proteins, with a total of 39 (78) transitions. Numbers in parenthesis include heavy isotope peptide standards. Three proteins were represented by two peptides showing correlations (R^2) of 0.689-0.842 and interassay CV values for seven peptides were less than 9 % (ESM Table 1). All sample measurements were within the linear part of the standard curves (ESM Fig. 1) and above the lower limit of quantification (ESM Table 1).

Supporting references

[1] Overgaard M, Cangemi C, Jensen ML, Argraves WS, Rasmussen LM (2015) Total and isoform-specific quantitative assessment of circulating fibulin-1 using selected reaction monitoring MS and time-resolved immunofluorometry. Proteomics Clin Appl 9:767-775

[2] McNulty DE, Annan RS (2008) Hydrophilic interaction chromatography reduces the complexity of the phosphoproteome and improves global phosphopeptide isolation and detection. Mol Cell Proteomics 7 (5):971-980

[3] Melo-Braga MN, Schulz M, Liu Q, Swistowski A, Palmisano G, Engholm-Keller K, Jakobsen L, Zeng X, Larsen MR (2014) Comprehensive quantitative comparison of the membrane proteome,

phosphoproteome, and sialiome of human embryonic and neural stem cells. Mol Cell Proteomics 13(1): 311-328.
