# Supplementary material for: First-trimester proteomic profiling identifies novel predictors of gestational diabetes mellitus
Source: PLoS One. 2019 Mar 27;14(3):e0214457. doi: 10.1371/journal.pone.0214457 (PMC6436752; doi:10.1371/journal.pone.0214457)
Supplement: S1 Fig — (PDF) [file pone.0214457.s002.pdf]

S1 Fig. Linearity of dilution of stable isotope labelled standard peptides

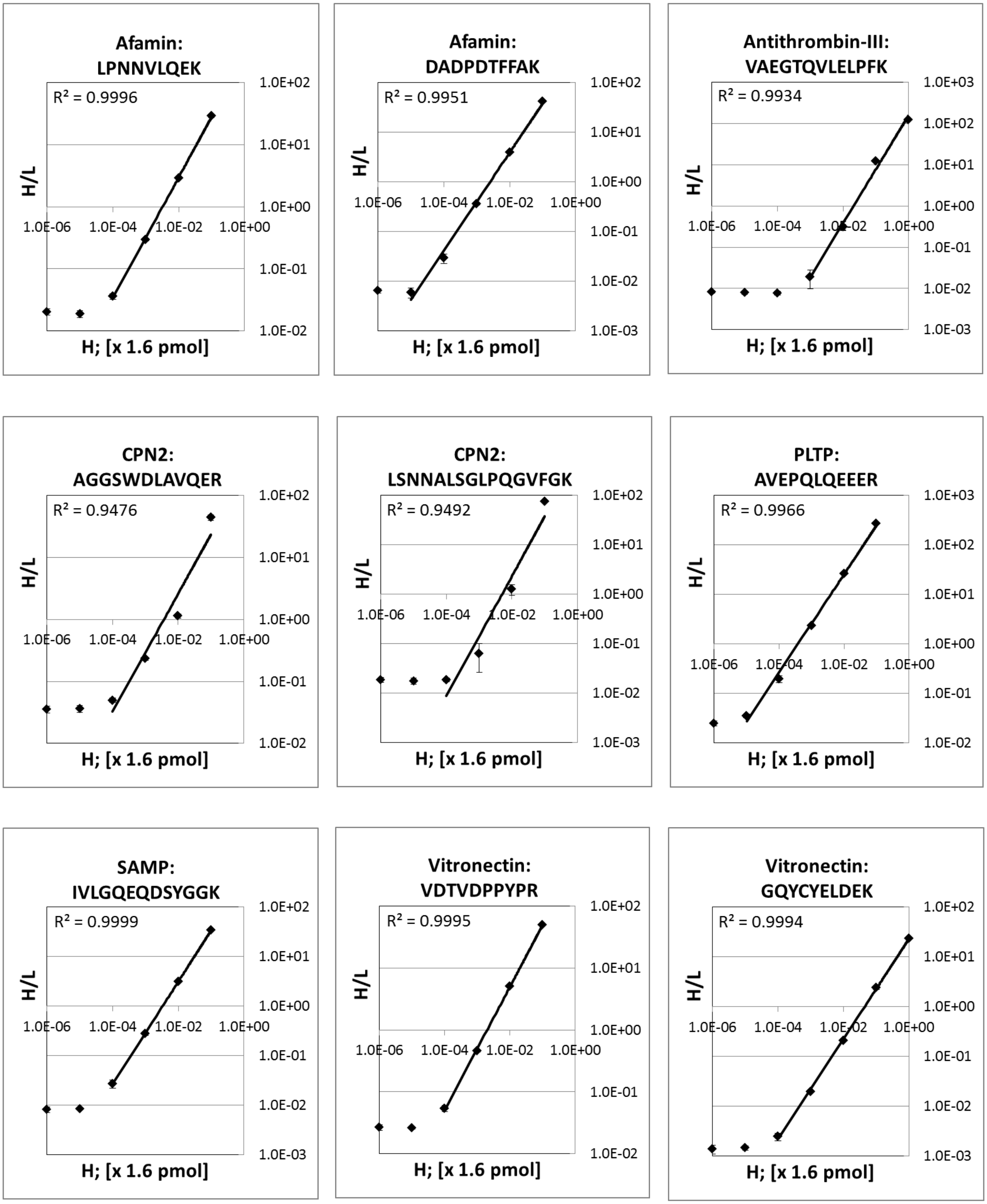

ESM Fig. 1 Standard curves of the MRM-MS assay for all 9 peptides included. The curves shows a linear relationship between the different amounts (not absolute) of heavy labelled standard peptide [H], spiked in to a constant serum pool, and the MS signal, measured as the signal ratio of heavy labelled standard peptide to endogenous light peptide (H/L). CPN2: Carboxypeptidase N subunit 2, PLTP: Phospholipid transfer protein, SAMP: Serum amyloid P-component.
