# Supplementary material for: First-trimester proteomic profiling identifies novel predictors of gestational diabetes mellitus
Source: PLoS One. 2019 Mar 27;14(3):e0214457. doi: 10.1371/journal.pone.0214457 (PMC6436752; doi:10.1371/journal.pone.0214457)
Supplement: S2 Fig — (PDF) [file pone.0214457.s003.pdf]

**S2 Fig. Linear correlation of tryptic peptides for quantitation of afamin, vitronectin and CPN2.**

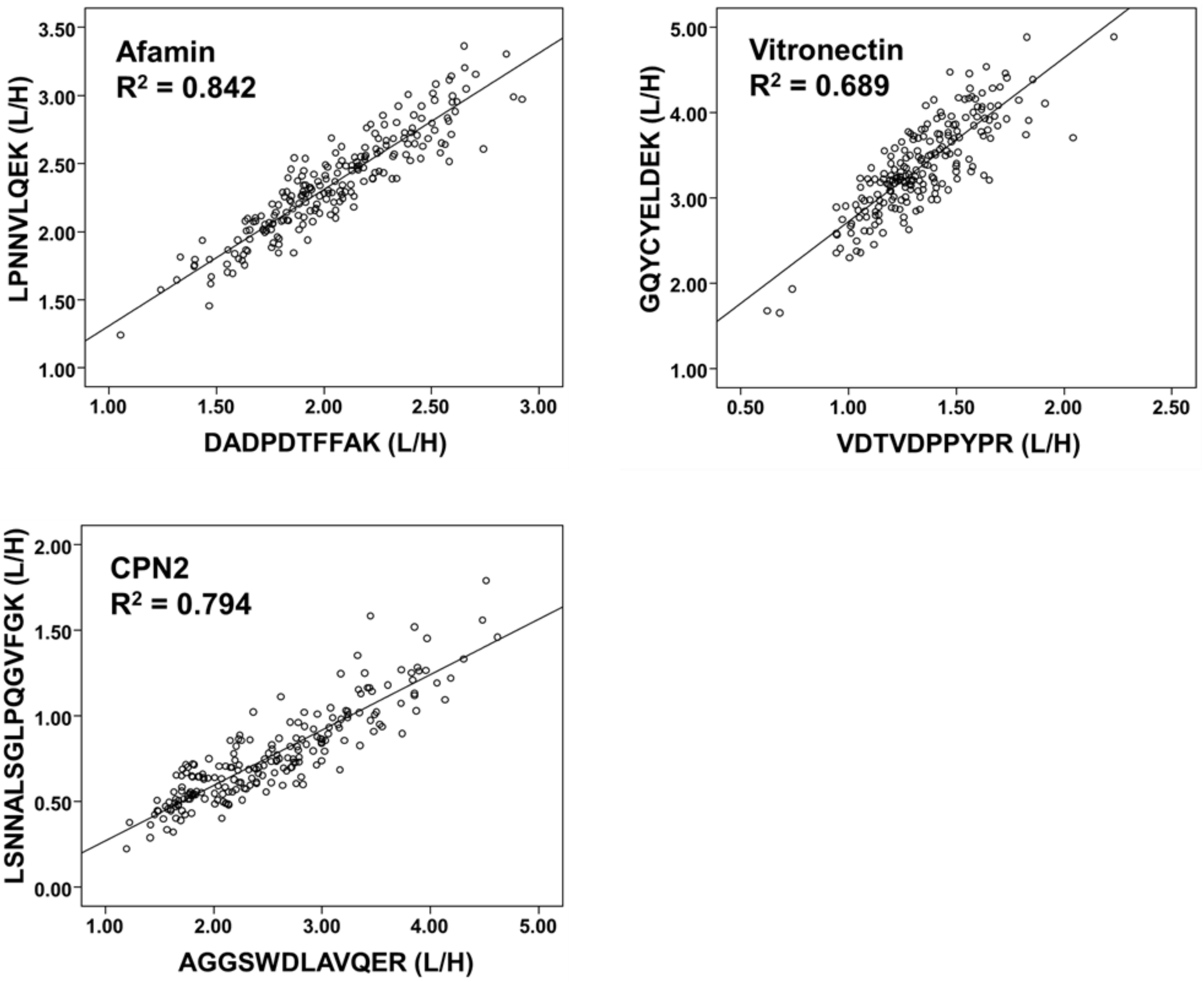


**ESM Fig. 2** Scatterplots of all 210 data points from the MRM-MS assay, showing a linear correlation between the ratio of endogenous light peptide to spiked in heavy labelled peptide (L/H) of the two peptides representing each of the proteins afamin, vitronectin and Carboxypeptidase N subunit 2 (CPN2) with a squared correlation coefficient ($R^2$) of 0.842, 0.689 and 0.794 respectively.
