# Supplementary material for: First-trimester proteomic profiling identifies novel predictors of gestational diabetes mellitus
Source: PLoS One. 2019 Mar 27;14(3):e0214457. doi: 10.1371/journal.pone.0214457 (PMC6436752; doi:10.1371/journal.pone.0214457)
Supplement: S1 Table — (PDF) [file pone.0214457.s004.pdf]

S1 Table. MRM 6-plex assay

Protein	Precursor[a]	Product ions[b]	CV %[c]	LLOQ[d] (fmol)
Afamin			3.5	
	LPNNVLQEK	y3, y4, y5, y7	3.8	0.16
	DADPDTFFAK	y5, y6, y7, y8	4.6	0.016
Antithrombin-III			8.9	
	VAEGTQVLELPFK	y4, y5, y6, y7, y10	8.9	1.6
Carboxypeptidase N subunit 2			21.5	
	AGGSWDLAVQER	y3, y4, y5, y6	22.4	0.16
	LSNNALSGLPQGVFGK	y5, y7, y9, y10, y11	20.4	0.16
Phospholipid transfer protein			5.0	
	AVEPQLQEEER	y4, y5, y8, y9	5.0	0.016
Serum amyloid P-component			4.0	
	IVLGQEQDSYGGK	y5, y6, y8, y10, y11	4.0	0.16
Vitronectin			3.8	
	VDTVDPPYPR	y4, y5, y6	6.1	0.16
	GQYCYELDEK	y4, y5, y6, y7, y8	2.6	0.16

[a]Precursor ions have charge +2

[b]Product ions have charge +1

[c]Interassay CV

[d]LLOQ, lower limit of quantitation on column, lowest linear response as derived from Fig S1
