# Supplementary material for: First-trimester proteomic profiling identifies novel predictors of gestational diabetes mellitus
Source: PLoS One. 2019 Mar 27;14(3):e0214457. doi: 10.1371/journal.pone.0214457 (PMC6436752; doi:10.1371/journal.pone.0214457)
Supplement: S3 Table — (PDF) [file pone.0214457.s006.pdf]

S3 Table. Binominal logistic regression analysis

Model	Variables included in model	**Validation set (*n* = 210)**	
		**AUC**	***p* of AUC**
	PrevGDM	0.583 (0.504, 0.661)	0.043
1	Mage	0.638 (0.563, 0.714)	0.001
2	FamDiab	0.707 (0.635, 0.780)	3.9×10^{-7}
	PrevGDM + Mage[a]	0.682 (0.608, 0.755)	9.0×10^{-6}
	PrevGDM + FamDiab[a]	0.771 (0.704, 0.837)	3.5×10^{-11}
3	Mage[a] + FamDiab[a]	0.798 (0.736, 0.861)	2.7×10^{-13}
	PrevGDM + Mage[a] + FamDiab[a]	0.828 (0.770, 0.886)	9.3×10^{-16}
A	SAMP	0.581 (0.504, 0.658)	0.042
B	Afamin	0.612 (0.536, 0.689)	0.005
C	Vitronectin	0.625 (0.550, 0.701)	0.002
1.A	Mage[a] + SAMP[a]	0.659 (0.585, 0.733)	6.8×10^{-5}
1.B	Mage[a] + afamin[a]	0.676 (0.603, 0.748)	1.1×10^{-5}
1.C	Mage[a] + vitronectin[a]	0.681 (0.609, 0.753)	6.0×10^{-6}
2.A	FamDiab[a] + SAMP	0.750 (0.682, 0.818)	9.4×10^{-10}
2.B	FamDiab[a] + afamin	0.715 (0.643, 0.787)	1.4×10^{-7}
2.C	FamDiab[a] + vitronectin[a]	0.760 (0.695, 0.826)	1.8×10^{-10}
3.A	Mage[a] + FamDiab[a] + SAMP	0.803 (0.741, 0.864)	1.2×10^{-13}
3.B	Mage[a] + FamDiab[a] + afamin	0.799 (0.737, 0.861)	2.4×10^{-13}
3.C[b]	Mage[a] + FamDiab[a] + vitronectin[a]	0.806 (0.746, 0.867)	6.3×10^{-14}

[a] Variables that contributes significantly to the model (p <0.05).

[b] Model also achieved by logistic regression of the 6 variables; Previous GDM (PrevGDM), Maternal age (Mage), Family history of diabetes (FamDiab), SAMP, afamin and vitronectin, removing the variable with least contribution to the model until all remaining variables contributed significantly.

Models including PrevGDM and FamDiab comprise n = 201 due to missing data.
